# Comparison between Two Low Doses of Amitriptyline in the Management of Chronic Neck Pain: A Randomized, Double-Blind, Comparative Study

**DOI:** 10.1155/2021/8810178

**Published:** 2021-01-19

**Authors:** Atef Mohamed Sayed Mahmoud, Safaa Gaber Ragab, Maged Labib Boules, Joseph Makram Botros

**Affiliations:** Pain and Intensive Care Medicine, Faculty of Medicine, Fayoum University, Fayoum, Egypt

## Abstract

Chronic neck pain (CNP) is a major concern for pain therapists. Many drugs including antidepressants such as amitriptyline have been used in the management of CNP. This study compared the efficacy and safety of 2 different doses of amitriptyline (5 mg and 10 mg at bedtime) in patients with CNP. A total of 80 patients of both sexes with idiopathic CNP, ranging in age from 18 to 75 years, were divided into 2 groups that received 5 or 10 mg oral amitriptyline at bedtime for 120 days. The primary outcome measure was neck pain disability index (NPDI). Neck pain intensity, Athens Insomnia Scale score, Hospital Anxiety and Depression Scale (HADS), side effects of the drug, and patient satisfaction were secondary outcome measures. NPDI decreased by 71.9% ± 13.4% in the 10 mg group compared to 47.3% ± 17.3% in the 5 mg group, representing a statistically significant difference (95% confidence interval: 27.3–12.6). Additionally, the 10 mg group showed greater mean reductions in pain score and HADS scores (both the anxiety and depression subscales), as well as improvement in sleep disturbance compared to the 5 mg group. A higher dose (10 mg) of amitriptyline at bedtime significantly reduced neck pain intensity, sleep disturbance, and anxiety and depression compared to a lower dose (5 mg) in patients with idiopathic and nontraumatic CNP after 120 days of treatment, with no significant difference between groups in the rate of side effects.

## 1. Introduction

Although idiopathic chronic neck pain (CNP) is a common and debilitating condition, its management is not always optimal. CNP is a musculoskeletal disorder characterized by continuous or recurrent pain lasting at least 3 months [1] that affects 10%–20% of the general population and is more common in women and people affected by psychosocial stress, specific neck and shoulder injuries, and repetitive physical strain, with a peak prevalence between the ages of 55 and 64 years [1]. CNP has a considerable socioeconomic impact and is associated with disability and reduced quality of life in patients [2, 3].

CNP has been shown to be associated with increased sympathetic activity [4]; abnormal changes in heart rate during sleep reflecting autonomic dysregulation were observed among workers with chronic neck and shoulder pain [5], with similar findings reported in individuals with CNP [6, 7]. Most individuals with CNP have no history of trauma but biomechanical, psychological, and cognitive factors can induce a shift from acute to CNP [1, 6, 8]. Chronic musculoskeletal pain has different underlying mechanisms including amplification of nociceptive input from myofascial A-delta and C fibers, second-order spinal neuron sensitization, stimulation of supraspinal neurons that facilitates pain conduction, and decreased activation of descending antinociceptive pathways [9]. These mechanisms are likely interrelated, as central sensitization can be achieved by sustained nociceptive inputs from peripheral myofascial tissues [10].

Nonsteroidal anti-inflammatory drugs, acetaminophen, and muscle relaxants have been shown to be effective in the management of acute neck pain; however, they rarely provide adequate pain relief in CNP. Thus, there are currently no effective treatments for CNP [11]. Chronic pain and depression are highly prevalent conditions with overlapping symptoms, with numerous studies reporting a reciprocal association between emotional disorder (especially depression) and pain [12, 13].

Although evidence-based strategies are used for the clinical management of CNP, a standardized treatment protocol is lacking [14, 15]. Tricyclic antidepressants (TCAs) are recommended for the management of some chronic pain syndromes including chronic tension-type headache (CTTH), fibromyalgia, and neuropathic pain [16]. These disorders, especially CTTH, are associated and even share clinical and pathophysiologic features with CNP, suggesting that they can be effectively managed using the same strategies [1, 16–18].

Amitriptyline was previously shown to be the most effective antidepressant for the treatment of CTTH [19] and was found to be superior to other drugs in preventing migraine [20]. Although amitriptyline has multiple undesirable effects at high doses, these are minimized at lower doses and gradually disappear over time, although its analgesic effects are long-lasting [18, 19]. Consequently, this drug has frequently been used to manage CNP even in the absence of evidence of its efficacy and safety from clinical trials [17], although a low dose (5 mg) of amitriptyline was found to be effective in alleviating idiopathic CNP with fewer side effects compared to a placebo [2].

This prospective, randomized, double-blind comparative study was carried out in order to compare the efficacy and tolerability of low doses of amitriptyline (5 and 10 mg) in patients with idiopathic CNP.

## 2. Materials and Methods

### 2.1. Patients

The study protocol was approved by the Institutional Ethics Committee of El Fayoum University Hospitals. A total of 80 patients of both sexes with idiopathic CNP, ranging in age from 18 to 75 years, were recruited at 2 centers: the Pain Outpatient Clinic at Al-Fayoum University Hospitals and a private pain clinic in El-Fayoum City, Egypt. The study has been registered in the Pan African Clinical Trial Registry (identification number PACTR202003721200086).

Patients underwent both general and neurologic examinations, which were performed by the same neurologist and pain consultants. Cervical spine radiographs (anteroposterior and lateral views) were obtained for all patients during their first visit and were evaluated by the same 2 radiologists. Patients with CNP for more than 15 days per month and lasting at least 3 months, without a history of trauma or other neurologic disorders, with normal neurologic function (as assessment by a neurologist), and without any abnormalities on imaging except for a loss of cervical lordosis were included.

**Figure 1:**
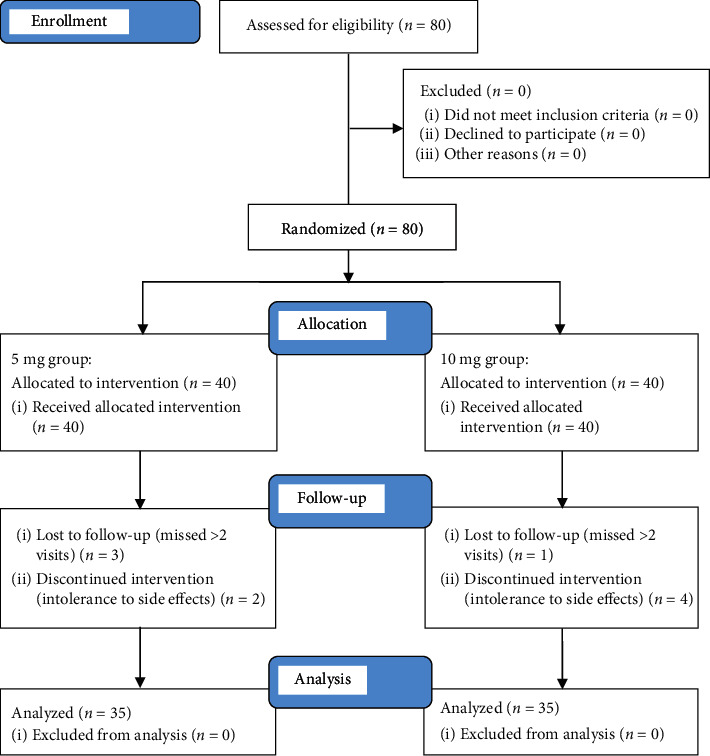
CONSORT flow diagram of patient selection.

Patients who had any other neurologic conditions; had abnormal imaging findings; had a history of cervical disc disease, migraine headaches, trauma, or major depressive disorder; abused analgesics or experienced side effects of tricyclic or tetracyclic antidepressants; had existing psychiatric illnesses or a history of glaucoma, arrhythmia, or severe constipation; or were taking medications for CNP (except nonsteroidal anti-inflammatory drugs or paracetamol) in the preceding month; as well as pregnant women and patients with prostatic symptoms were excluded. Neck pain was defined as pain in the posterior aspect of the neck and anatomical projection to the trapezius muscle, sometimes involving the head without any arm pain. A flow diagram of the patient selection process is shown in Figure 1.

### 2.2. Study Design

The study was designed as a double-blind, prospective, comparative, randomized clinical trial. Patients who met the inclusion and exclusion criteria were provided with a protocol information document and consent form stating that they would receive either 5 or 10 mg of amitriptyline. Enrolled patients were allocated (by computer-generation block randomization) to either the 5 or 10 mg amitriptyline groups (*n* = 40 patients per group). The patients were given 120 pills of 5 or 10 mg amitriptyline in a box, to be taken at bedtime. To ensure double-blinding, neither the patients nor the attending physician was aware of group assignment. No extra medications were allowed during the 4-month trial.

Compliance with the drug regimen was monitored at visits to the outpatient clinic every 2 weeks by counting the number of amitriptyline pills in each patient's box. Patients who experienced side effects were considered intolerant to the drug and allowed to discontinue treatment and were excluded from the analysis. The final assessment was performed at the end of the 4-month trial period by a senior resident who was blinded to group allocation.

### 2.3. Clinical Assessment and Measured Parameters

Clinical assessment of the patients was performed at enrollment and at the end of the 4-month trial. The primary outcome measure was the neck pain disability index (NPDI), which reflects disability secondary to pain [21]. The NPDI questionnaire was adapted and validated for the Egyptian population and comprised 10 items, each scored from 0 to 5 for a maximum score of 50. The original index had the following scoring intervals: 0–4 = no disability; 5–14 = mild disability; 15–24 = moderate disability; 25–34 = severe disability; >34 = very severe disability.

Secondary outcome measures were also evaluated before and at the end of the 4-month treatment period. Neck pain intensity was measured using the visual analog scale (VAS), with 0 indicating no pain and 10 indicating the worst pain imaginable. Insomnia was assessed using the Athens Insomnia Scale (AIS), which is a self-administered test with symptom-related questions [22]. Anxiety and depression symptoms were evaluated with the Hospital Anxiety and Depression Scale (HADS), with scores from 0 to 21 [23].

Side effects of the 2 doses of amitriptyline were measured and recorded. At the end of the 4-month trial, the patient's subjective satisfaction was measured on a scale from 0 to 10, with 0 indicating complete dissatisfaction and 10 indicating complete satisfaction. Patients missing more than 5 out of the 120 days of treatment were excluded from the analysis.

### 2.4. Sample Size Calculation

The sample size for this trial was calculated using a power of 80% and 5% level of error. The mean percent improvement in the primary outcome measure (NPDI) with 5 mg amitriptyline was 42% ± 15% in a previous study [2]. An additional 10% improvement with a higher dose (10 mg) was considered satisfactory. The minimum calculated sample size was 35 per group based on the following equation: *n*1= (*σ*12 + *σ*22/*K*) (*z*1 − *α*/2 + *z*1−*β*)2/Δ2. We therefore recruited 40 patients per group, increasing the sample size by 12% to compensate for potential dropouts.

### 2.5. Statistical Analysis

Demographic and baseline characteristics of the patients were summarized as means and standard deviations for numeric data and as frequency distributions for categorical data. Data for the 2 treatment groups were analyzed for any imbalances after randomization. The primary analysis included comparisons of the main outcome measure (improvement in NPDI after 4 months of treatment) between groups using the independent samples *t*-test, which was also used to analyze secondary outcomes. The difference in the rate of side effects in each group was evaluated with the chi-squared test. The correlation between patient satisfaction and other outcomes was assessed with Pearson's correlation coefficient. Statistical significance was set at *P* < 0.05. Data were analyzed with SPSS v24 software (IBM, Armonk, NY, USA).

## 3. Results

### 3.1. Baseline Characteristics of the Study Population

A total of 80 patients were enrolled in the study, with 40 patients each receiving 5 and 10 mg amitriptyline for 4 months. Ten patients were excluded from the final assessment because of intolerance to side effects or loss to follow-up. Baseline patient characteristics and demographic data were comparable between the 2 groups (Table 1). At baseline, there were no differences in age, body mass index, NPDI, pain score, AIS, and HADS anxiety and depression subscales (HAD-A and -D, resp.) between the 2 groups (Table 2).

AIS, Athens Insomnia Scale; BMI, body mass index; HAD-A, Hospital Anxiety and Depression Scale-anxiety subscale; HAD-D, Hospital Anxiety and Depression Scale-depression subscale; NPDI, neck pain disability index.

### 3.2. Effects of Amitriptyline on CNP and Other Symptoms

**Figure 2:**
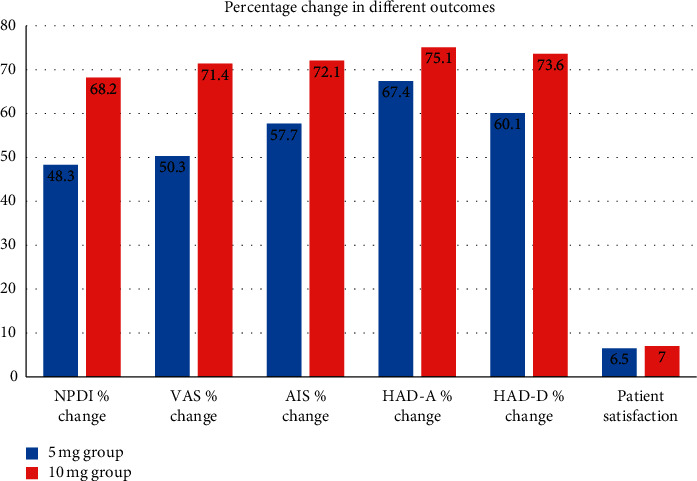
Changes in outcome measures following treatment with amitriptyline.

The changes in outcome measures following treatment with amitriptyline are shown in Figure 2. NPDI, the primary outcome measure, decreased by 71.9% ± 13.4% in the 10 mg group, which was greater than the decrease in the 5 mg group (47.3% ± 17.3%) (*P* < 0.001). The mean reduction in pain scores was 5.7 ± 1.4 in the 10 mg group and 3.8 ± 1.2 in the 5 mg group, representing a statistically significant difference. Similarly, VAS decreased to a greater extent in the 10 mg group than in the 5 mg group (71.4% ± 13.7% vs 50.3% ± 15.9%) (Table 3).

AIS, Athens Insomnia Scale; HAD-A, Hospital Anxiety and Depression Scale-anxiety subscale; HAD-D, Hospital Anxiety and Depression Scale-depression subscale; NPDI, neck pain disability index; VAS, visual analog scale.

The higher dose of amitriptyline yielded a greater improvement in sleep, as measured by the AIS (72.1% ± 12% for 10 mg group vs 57.7% ± 10.2% for 5 mg group) (Table 3). Treatment with 10 mg amitriptyline also led to a greater decrease in HADS scores compared to the 5 mg dose (HAD-A: 75.1% ± 12% vs 67.4% ± 7.1%; HAD-D: 73.6% ± 8.8% vs 60.1% ± 7.4%) (*P* < 0.05 for both). There was no difference in patient satisfaction between the 2 groups at the end of the treatment period.

There were no significant differences in the rate of complications between the 2 groups (Table 4). NPDI and other outcome measures showed a strong positive correlation with VAS; weak positive correlations with AIS, HDAD-A, and HAD-D; and no correlation with patient satisfaction (Table 5). Meanwhile, VAS and other outcome measures showed a strong positive correlation with NPDI, moderate positive correlation with HAD-D, weak positive correlations with HAD-A and AIS, and no significant correlation with patient satisfaction (Table 5).

## 4. Discussion

Antidepressants have been shown to be effective for pain management; for example, the selective serotonin reuptake inhibitor (SSRI) paroxetine has been used to treat fibromyalgia [24], while the SSRI zimelidine significantly alleviated chronic pain [25]. A recent review reported that SSRIs such as fluoxetine, fluvoxamine, and escitalopram were effective in the treatment of chronic pain [26]. TCAs are not recommended in current international guidelines for the management of chronic pain, but low-dose amitriptyline may be effective based on its action in other pain syndromes [27, 28] such as chronic facial pain and somatic pain (including CNP and different types of chronic headache); and many patients favor TCAs for their availability, low cost, and effectiveness [18, 29].

In clinical practice, it is important to establish the minimum effective dose of a drug to reduce side effects. To this end, in the present study we compared the efficacy of 2 different doses of amitriptyline for the treatment of idiopathic and nontraumatic CNP. The results showed that the higher of the 2 doses (10 mg) relieved pain and improved emotional disorder (anxiety and depression) to a greater extent than the lower dose (5 mg) without increasing the occurrence of side effects.

The mechanism of action of amitriptyline in CNP is not known, as this drug exerts multiple effects on the nervous system and the pathophysiology of CNP is unclear. Animal studies have shown that amitriptyline may antagonize the N-methyl-d-aspartate receptor, thereby inhibiting the firing of second-order neurons [30]. Additionally, it has been suggested that amitriptyline inhibits serotonin and norepinephrine reuptake to enhance the action of endogenous opioids on descending antinociceptive pathways, leading to pain suppression [17]. A previous study examining the effects of 25 mg amitriptyline on chronic back pain found a significant improvement in disability at 3 months with minimal adverse events [31], and a recent meta-analysis showed that amitriptyline is also an effective prophylactic against migraine [20].

We used the NPDI as the primary outcome measure, as most of our patients were workers who were functionally incapacitated in their work as a result of CNP. NPDI improved to a greater extent with 10 mg amitriptyline than with the 5 mg dose. In a previous study, 5 mg amitriptyline decreased NPDI by 42.22% ± 15.5% in patients with CNP [2], which is comparable to the improvement observed in our patients who were treated with the same dose (47.3% ± 17.3%).

The mean decreases in VAS and pain score and improvement in AIS were greater with 10 mg amitriptyline as compared to the 5 mg dose. The mean percent reduction in VAS in the 5 mg group in our study (50.3% ± 15.9%) was also similar to the previously reported value [2], which lends validity to our results. Sleep disorders and chronic pain are mutually dependent [32, 33], and a significant correlation has been reported between the degree of sleep improvement and pain relief [2]. Low doses of amitriptyline may have a minor sedative effect that could alleviate sleep instability in addition to reducing pain intensity. In our study, AIS score increased by 57.7% ± 10.2% in the 5 mg amitriptyline group, which is higher than the previously reported improvement of 34.89% ± 22.98% [2]. This discrepancy may be attributable to the different instruments that were used; Maarrawi et al.[2] used the Bergen Insomnia Scale (BIS) while we used the AIS, which we found easier to administer to our patients. Additionally, differences in cultural background, social class, and education level between the 2 study populations may have contributed to the divergent findings.

Anxiety and depression in our CNP patients, as evaluated by HAD-A and -D, respectively, were improved to a greater extent by 10 mg as compared to 5 mg amitriptyline. However, despite these and other positive effects associated with the higher drug dose, there was no difference in the level of satisfaction between the 2 groups for reasons that remain unclear. We observed positive correlations between VAS and HAD-A and -D scores in patients treated with 10 mg amitriptyline, although the correlations were weak and moderate, respectively. In the study by Maarrawi et al., there was no correlation between the primary outcome measure for CNP (VAS) and anxiety and depression scores [2], suggesting that the positive effects of amitriptyline on pain and emotional disorder occur via distinct mechanisms. This is supported by the observation that the SSRI sertraline improved the emotional state of patients with pelvic pain while having no clinically meaningful effect on pain intensity [34].

## 5. Conclusions

A higher dose of amitriptyline (10 mg) was more effective than a lower dose (5 mg) in alleviating neck pain, disability, sleep problems, and anxiety and depression symptoms in patients with idiopathic and nontraumatic CNP, without increasing the rate of adverse effects. Based on these findings, we recommend 10 mg amitriptyline for the clinical management of CNP.

## Figures and Tables

**Table 1:** Demographic and baseline characteristics of the study participants.

	Group		Total	*P* value^†^
	5 mg	10 mg		
Sex				1.000
F	26 (74.3)	26 (74.3)	52 (74.3)	
M	9 (25.7)	9 (25.7)	18 (25.7)	

Occupation				0.882
Doctor	0 (0.0)	1 (2.9)	1 (1.4)	
Engineer	1 (2.9)	1 (2.9)	2 (2.9)	
Farmer	8 (22.9)	8 (22.9)	16 (22.9)	
Healthcare worker	9 (25.7)	7 (20.0)	16 (22.9)	
Laboratory technician	2 (5.7)	1 (2.9)	3 (4.3)	
Merchant	1 (2.9)	0 (0.0)	1 (1.4)	
Nurse	3 (8.6)	3 (8.6)	6 (8.6)	
Officer	2 (5.7)	2 (5.7)	4 (5.7)	
Retired	1 (2.9)	0 (0.0)	1 (1.4)	
Student	0 (0.0)	1 (2.9)	1 (1.4)	
Teacher	2 (5.7)	5 (14.3)	7 (10.0)	
Laborer	6 (17.1)	6 (17.1)	12 (17.1)	

Physical activity				0.955
High	10 (28.6)	9 (25.7)	19 (27.1)	
Moderate	12 (34.3)	13 (37.1)	25 (35.7)	
Low	13 (37.1)	13 (37.1)	26 (37.1)	

Income level				0.580
High	1 (2.9)	2 (5.7)	3 (4.3)	
Middle	10 (28.6)	13 (37.6)	23 (32.9)	
Low	24 (68.6)	20 (57.1)	44 (62.9)	

Duration of pain (months)				0.403
<3	5 (14.3)	3 (8.6)	8 (11.4)	
3–6	18 (51.4)	13 (37.1)	31 (44.3)	
6–12	8 (22.9)	12 (34.3)	20 (28.6)	
>12	4 (11.4)	7 (20.0)	11 (15.7)	

Loss of neck lordosis				0.632
Yes	18 (51.4)	16 (45.7)	34 (48.6)	
No	17 (48.6)	19 (54.3)	36 (51.4)	

ASA grade				0.919
I	19 (54.3)	20 (57.1)	39 (55.7)	
II	12 (34.3)	12 (34.3)	24 (34.3)	
III	4 (11.4)	3 (8.6)	7 (10.0)	

Data are expressed as *n* (%). ^†^Between 5 and 10 mg groups. ASA, American Society of Anesthesiologists.

**Table 2:** Select baseline demographic and clinical characteristics of patients.

	Group		*P* value^†^
	5 mg	10 mg	
Age	47.0 (11.0)	46.2 (10.7)	0.776
BMI	28.1 (3.1)	27.8 (3.2)	0.650
NPDI	28.5 (4.7)	29.1 (3.5)	0.547
Pain	7.6 (1.1)	7.9 (1.1)	0.230
AIS	12.6 (3.5)	13.3 (3.3)	0.418
HAD-A	12.2 (2.4)	12.4 (2.3)	0.650
HAD-D	10.2 (1.8)	10.7 (1.8)	0.247

Data are shown as mean (SD). ^†^Between 5 and 10 mg groups.

**Table 3:** Outcomes of patients with chronic neck pain treated with 5 and 10 mg amitriptyline.

	Group		*P* value	Mean difference	95% CI
	5 mg	10 mg			
NPDI change	48.3 (17.2)	68.2 (13.4)	<0.001^*∗*^	19.9	27.3, 12.6
Pain decrease	3.8 (1.2)	5.7 (1.4)	<0.001^*∗*^	1.9	2.5, 1.3
VAS % change	50.3 (15.9)	71.4 (13.7)	<0.001^*∗*^	21.1	28.2, 14.0
AIS % change	57.7 (10.2)	72.1 (12.0)	<0.001^*∗*^	15.4	20.7, 10.1
HAD-A % change	67.4 (7.1)	75.1 (6.9)	<0.001^*∗*^	7.7	11.1, 4.4
HAD-D % change	60.1 (7.4)	73.6 (8.8)	<0.001^*∗*^	13.6	17.4, 9.7
Patient satisfaction	6.5 (1.3)	7.0 (1.2)	0.117	0.5	1.1, −0.1

Data are expressed as *n* (%). ^*∗*^*P* < 0.05.

**Table 4:** Incidence of side effects in patients with chronic neck pain treated with 5 and 10 mg amitriptyline.

Side effect	Group		Total	*P* value
	5 mg	10 mg		
CVS				0.629
0	21 (60.0)	19 (54.3)	40 (57.1)	
1	14 (40.0)	16 (45.7)	30 (42.9)	

CNS				0.334
0	22 (62.9)	18 (51.4)	40 (57.1)	
1	13 (37.1)	17 (48.6)	30 (42.9)	

Anticholinergic				0.629
0	21 (60.0)	19 (54.3)	40 (57.1)	
1	14 (40.0)	16 (45.7)	30 (42.9)	

Allergic				1.000
0	28 (80.0)	28 (80.0)	56 (80.0)	
1	7 (20.0)	7 (20.0)	14 (20.0)	

Hematologic				0.780
0	27 (77.1)	26 (74.3)	53 (75.7)	
1	8 (22.9)	9 (25.7)	17 (24.3)	

GIT				0.454
0	24 (68.6)	21 (60.0)	45 (64.3)	
1	11 (31.4)	14 (40.0)	25 (35.7)	

Endocrine				0.110
0	31 (88.6)	25 (73.5)	56 (81.2)	
1	4 (11.4)	9 (26.5)	13 (18.8)	

Weight changes				0.434
0	26 (74.3)	23 (65.7)	49 (70.0)	
1	9 (25.7)	12 (34.3)	21 (30.0)	

Alopecia				0.101
0	32 (91.4)	27 (77.1)	59 (84.3)	
1	3 (8.6)	8 (22.9)	11 (15.7)	

0 = Absence of side effects; 1 = presence of side effects. CNS, central nervous system; CVS, cardiovascular system; GIT, gastrointestinal tract.

**Table 5:** Correlations between NPDI, VAS score, and other outcome measures.

Correlation with NPDI	Patient satisfaction	AIS	HAD-A	HAD-D	VAS
Pearson correlation coefficient	0.238^*∗*^	0.248^*∗*^	0.245^*∗*^	0.281^*∗*^	0.861^*∗∗*^
*P* Value	0.047	0.038	0.041	0.018	<0.001

Correlation with VAS	Patient satisfaction	AIS	HAD-A	HAD-D	NPDI

Pearson correlation coefficient	0.213	0.279^*∗*^	0.241^*∗*^	0.411^*∗∗*^	0.861^*∗∗*^
*P* Value	0.076	0.020	0.045	<0.001	<0.001

^*∗*^ *P* < 0.05. ^*∗∗*^*P* < 0.01. AIS, Athens Insomnia Scale; HAD-A, Hospital Anxiety and Depression Scale – anxiety subscale; HAD-D, Hospital Anxiety and Depression Scale-depression subscale; NPDI, neck pain disability index; VAS, visual analog scale.

## Data Availability

The data presented in this work are available from the corresponding author on reasonable request.
